# Severe capsular contracture in a patient with a history of multiple malignancies – Hematoma or neoplasm recurrence?: A case report

**DOI:** 10.1097/MD.0000000000039074

**Published:** 2024-08-02

**Authors:** Yutong Yuan, Fengzhou Du, Yiding Xiao, Jiuzuo Huang, Xiao Long

**Affiliations:** a Division of Plastic and Reconstructive Surgery, Peking Union Medical College Hospital, Beijing, China; b Department of Oncoplastic and Reconstructive Breast Surgery, Plastic Surgery Hospital, Chinese Academy of Medical Sciences and Peking Union Medical College, Beijing, China.

**Keywords:** breast cancer, breast reconstruction, capsule contracture, case report, complication

## Abstract

**Rationale:**

Complications associated with breast implants pose a significant obstacle to improving the quality of life for patients undergoing implant-based breast reconstruction. Due to the intricate nature of their presentation, diagnosis often becomes challenging and perplexing. Herein, we present a case report detailing the diagnostic and therapeutic processes employed in managing implant-related complications in a patient with multiple malignancies who underwent immediate breast reconstruction following mastectomy.

**Patent concerns:**

The patient, a 48-year-old woman, presented with severe pain and hardening in her left breast. She had previously undergone nipple-sparing mastectomy followed by immediate implant-based breast reconstruction 3 years ago.

**Diagnoses:**

Upon admission, we suspected a simple diagnosis of capsular contracture. However, upon investigation, she had a medical history of colon cancer, breast cancer, and acute B-lymphoblastic leukemia. Furthermore, she recently experienced nipple hemorrhage.

**Interventions:**

Considering her clinical manifestations, we postulated the possibility of tumor recurrence along with potential presence of breast implant-associated anaplastic large cell lymphoma. The situation took a new turn, as diagnostic imaging techniques including breast MRI, and ultrasound revealed indications of potential prosthesis rupture and periprosthetic infection.

**Outcomes:**

Ultimately, en bloc capsulectomy with implant removal was performed, revealing no evidence of implant rupture or infection but rather indicating delayed hematoma formation.

**Lessons:**

An accurate diagnosis of complications associated with breast prosthesis reconstruction is crucial for effective treatment. The examination and treatment processes employed in this case offer valuable insights toward achieving a more precise diagnosis of prosthesis-related complications, particularly in patients with complex medical histories.

## 1. Introduction

Implant-based breast reconstruction is the predominant surgical technique employed worldwide for breast cancer patients who have undergone nipple-sparing mastectomy.^[1]^ However, complications arising from immediate implant-based breast reconstruction after nipple-sparing mastectomy can significantly impact the quality of life of patients. The potential complications include hematoma, seroma, infection, capsular contracture, implant rupture, and prosthesis-related malignancies, such as breast implant-associated anaplastic large cell lymphoma (BIA-ALCL), breast implant-associated squamous cell carcinoma (BIA-SCC), and breast implant-associated diffuse large B-cell lymphoma (BIA-DLBCL).^[2–5]^ The occurrence of these complications is not uncommon, as evidenced by relevant literature indicating that the hematoma incidence in postmastectomy implant-based breast reconstruction ranges from 4% to 9%.^[6]^ However, the incidence rate of capsular contracture varies significantly across different sources due to factors such as radiotherapy, prosthesis placement level, and types of prostheses used. It typically falls within a range of approximately 7.5% to 47.5%.^[7–10]^ The prevalence of BIA-ALCL also exhibits considerable variation depending on the denominator employed. For patients with macrotextured implants, it has been observed to be as high as 1:300 in aesthetic augmentations and 1:350 in reconstructive cases.^[11,12]^ The absence of accurate clinical diagnoses for implant-related complications poses challenges in providing appropriate treatment for these patients. Herein we present a case study outlining the diagnostic and treatment process for a patient undergoing immediate breast implant reconstruction following nipple-sparing mastectomy with inconsistent and intricate diagnostic process. Our aim is to make a contribution toward enhancing diagnosis and management strategies for complications arising from prosthetic breast reconstruction.

## 2. Case report

**Figure 1.**
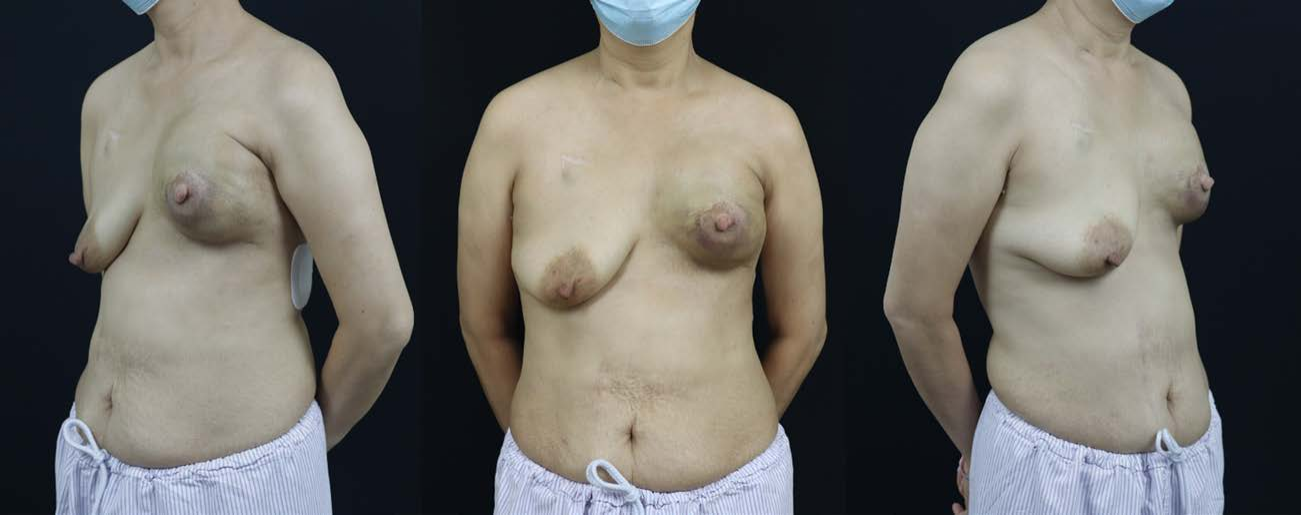
Preoperative general photographs of the breast region.

**Figure 2.**
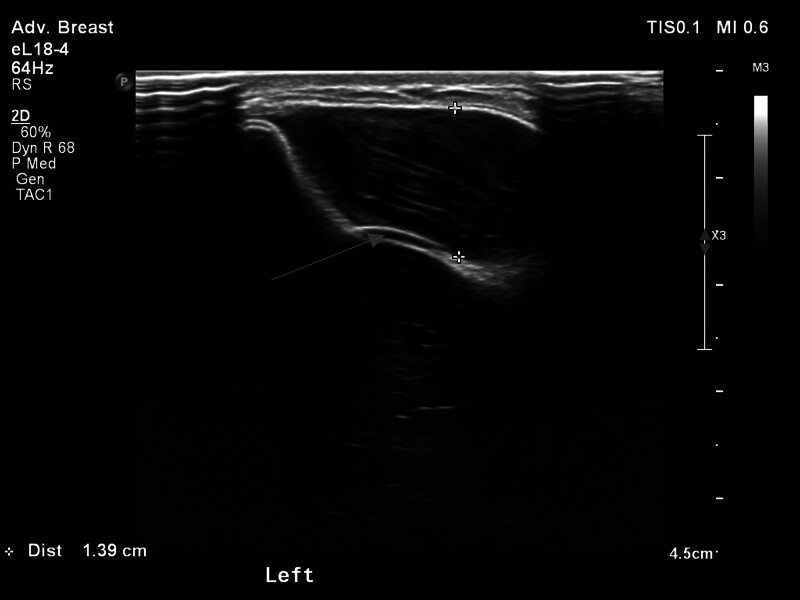
Breast ultrasound revealed capsule morphology with a surrounding hypoechoic fluid collection, accompanied by suspicious high echogenic linear signal beneath the capsule (indicated by the red arrow).

**Figure 3.**
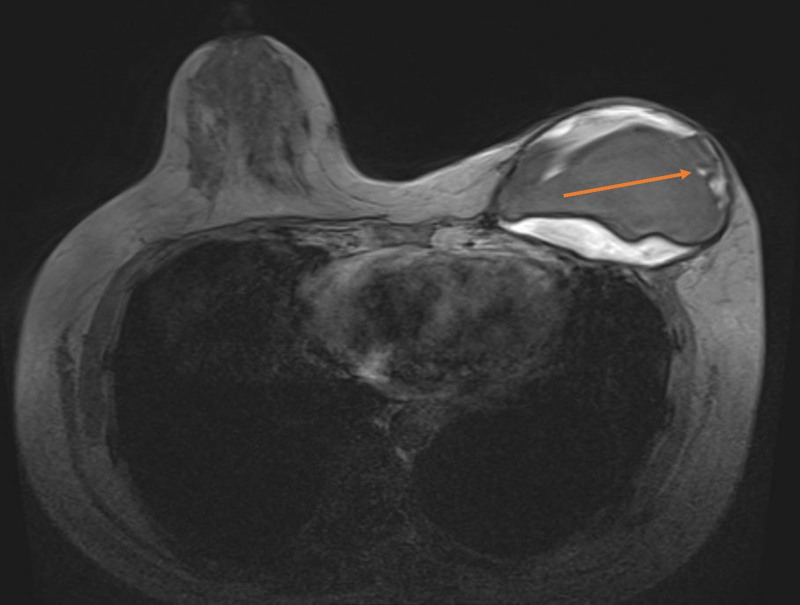
Breast MRI revealed fibrous capsule contracture surrounding the left mammary prosthesis, intracapsular effusion, suspicious silicone signal adjacent to the capsule, and rupture of the prosthesis shell, a suspicious tear drop sign can be observed (indicated by the red arrow). MRI = magnetic resonance imaging.

**Figure 4.**
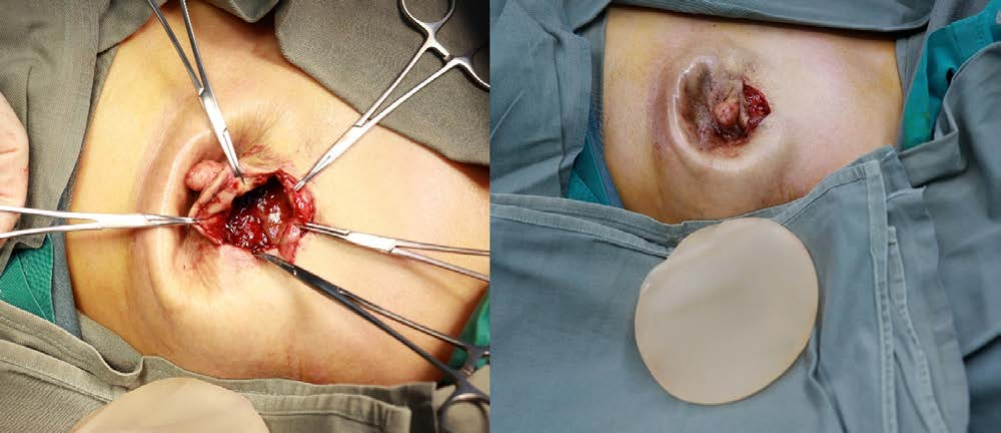
Intraoperatively, the prosthesis was observed to be intact, accompanied by a substantial presence of aged congestion and necrotic tissue within the capsule.

**Figure 5.**
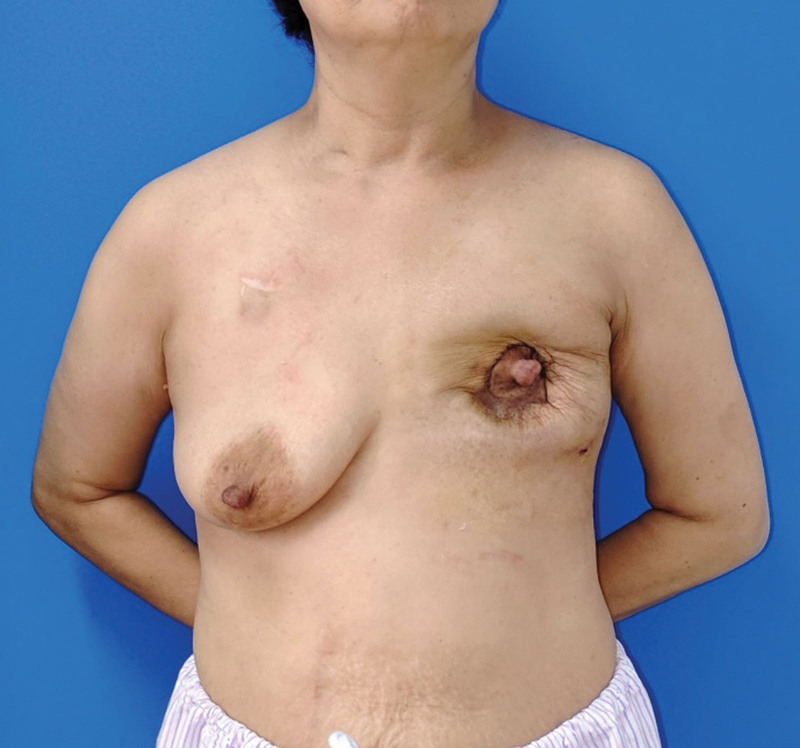
Postoperative general photographs of the breast region.

Our report is based on the CASE REPORT (CARE) guideline. The patient, a 48-year-old female, underwent nipple-sparing mastectomy followed by immediate subpectoral implant breast reconstruction at another medical facility 3 years ago. The round prosthesis featured textured surface, which did not incorporate an acellular dermal matrix or a mesh covering. However, due to the patient’s surgery being performed at another facility, we were unable to obtain additional details regarding the manufacturer of the prosthesis. She was admitted to our hospital due to intractable pain, which commenced 6 months ago and progressively exacerbated. After admission, the patient underwent a physical examination which revealed ecchymosis on the surface of the left breast, elevated skin tension, and palpable induration of the breast (Fig. 1). Based on her clinical presentation, we deduced that she was diagnosed with a simple Baker grade IV capsule contracture. However, upon thorough investigation, we discovered that she had experienced an episode of spontaneous nipple hemorrhage 2 months ago, and she denied any history of breast trauma. Moreover, she had a complex medical history encompassing multiple malignancies. Prior to the treatment of breast cancer, she was diagnosed with colorectal cancer and underwent radical right hemicolectomy. Following the breast reconstruction surgery, she developed acute B-lymphoblastic leukemia and received 7 cycles of chemotherapy. Currently, she is undergoing oral flumatinib maintenance therapy. Considering her history of nipple hemorrhage and multiple malignancies, we posited a potential tumor recurrence following the nipple-sparing mastectomy and the possibility of BIA-ALCL. Further, the obtained imaging findings complicated the patient’s diagnostic process. Breast ultrasound demonstrated undulating capsule morphology with a surrounding hypoechoic fluid collection and suspicious high echogenic linear signal beneath the capsule (Fig. 2). And the preoperative magnetic resonance imaging (MRI) revealed fibrous capsule contracture around the left mammary prosthesis, intracapsular effusion, suspicious silicone signal adjacent to the capsule, and rupture of the prosthesis shell (Fig. 3). And the results of positron emission tomography-computed tomography (PET-CT) indicated irregularity of the left breast prosthesis with periprosthetic effusion and increased metabolic activity in surrounding tissues and adjacent chest wall suggestive of possible implant rupture with infection. This result temporarily excluded the possibility of breast cancer recurrence. While due to the additional diagnosis of infection, we conducted hemogram and inflammatory indicator tests, which revealed an elevation in the levels of inflammatory markers and white blood cell. The erythrocyte sedimentation rate was measured at 51 mm/h, while hypersensitive C-reactive protein levels were found to be 16.43 mg/L. As for the complete blood cell count (CBC), white blood cell counts as 10.08 × 109/L, with neutrophil% at 58.4% and eosinophil% at 9.6%. Based on the patient’s clinical manifestations, radiographic findings, and blood test results prior to surgery, our preoperative diagnosis included prosthesis rupture, periprosthetic infection, and capsule contracture; Moreover, BIA-ALCL could not be excluded entirely. Consequently, we proceeded with left breast prosthesis removal and en bloc capsulectomy for this patient. Upon incising the capsule intraoperatively, it became evident that there was no prosthesis rupture; instead, a substantial presence of blood clot and necrotic tissue within the capsule was observed (Fig. 4). Etiological tests revealing no evidence of bacterial or fungal involvement in the etiology while pathological findings indicated necrotic tissue and capsule without any presence of tumor cells or CD30 positive leukomonocyte. Following a 7-day period of recovery and infection prevention, the inflammatory markers were reassessed and the patient was discharged. The patient felt relieved and less concerned about cancer without the reconstructed breast in his chest (Fig. 5).

## 3. Discussion

In general, the estimated lifespan of breast implants is approximately 10 years.^[13]^ Complications arising from prosthetic breast reconstruction following breast cancer surgery inflict pain upon numerous patients. The presence of mixed symptoms, signs, and laboratory findings hampers accurate diagnosis, thereby impeding appropriate follow-up treatment. In this study, we present a case of a breast reconstructed patient with multiple malignancies whose preoperative diagnosis did not align with the final clinical diagnosis. According to the patient’s performance, we initially diagnosed isolated severe capsular contracture. However, a thorough examination of the patient’s comprehensive medical history has raised concerns regarding potential recurrence of breast cancer or development of a new cancer. However, based on the findings derived from imageological examination, we began to strongly suspected that the patient was experiencing prosthesis rupture and periprosthetic infection. Ultimately, postoperative clinical assessment indicated varying complications: delayed hematoma formation. This infrequent occurrence of diagnostic discrepancies underscores the ongoing significance of investigating enhanced precision in clinical assessments of complications associated with breast prosthesis reconstruction.

Hematoma following prosthesis reconstruction primarily occurs in the early postoperative period.^[14]^ However, delayed hematoma occurrences, particularly 1 year after implantation, are exceedingly rare. Delayed hematoma is more commonly observed in patients with coagulation dysfunction or a history of trauma,^[15]^ and its diagnosis often relies on imaging examinations such as computed tomography (CT) scans, MRI scans, ultrasound evaluations along with pathological or cytological examination of fluid samples.^[16]^ Moreover, the presence of postoperative hematoma, as indicated in the relevant literature, serves as a risk factor for the development of capsular contracture following immediate implant-based breast reconstruction. This could potentially contribute to the severity of capsular contracture observed in this particular patient.^[17]^ The majority of prosthesis ruptures occur more than 6 years following implantation. Its diagnosis relies on patient-reported symptoms, including alterations in breast shape or firmness, breast pain as well as imaging techniques. Currently, magnetic resonance imaging plays a crucial role for detecting implant rupture. However, some studies indicate that MRI surveillance has a low sensitivity of implant rupture, while others suggest that the specificity of MRI is limited, with a false positive rate for implant rupture reaching up to 59%.^[18,19]^ Moreover, breast ultrasound can serve as a primary screening tool for identifying implant rupture. The newly ruptured prosthesis can exhibit inward folds in the ruptured shell, causing containment of overflowing contents within these folds and resulting in the tear drop sign. As prosthesis rupture progresses, separation occurs between the outer capsule and fibrous capsule, leading to the presence of high density lines beneath the capsule and even complete separation manifested as linguini sign.^[20]^ However, there was no definitive imaging evidence to confirm this diagnosis. Currently, diagnosis of BIA-ALCL relies on imaging investigations of suspicious masses or effusions along with immunohistochemical analysis, cytology examination, and flow cytometry assessment where CD30 expression is often strongly observed.^[21,22]^ Furthermore, although a case report has suggested the utilization of PET-CT for diagnosing and staging BIA-ALCL more accurately, there is currently insufficient empirical evidence to establish its feasibility at an evidence-based level.^[23]^

In this case, although the final procedure was appropriate (the patient had severe capsular contracture), considering a precise preoperative diagnosis, it may also be appropriate to perform en bloc capsulectomy and hematoma removal. Simultaneously, the risk stratification for BIA-ALCL and the assessment of potential risk factors for implant rupture holds paramount importance in terms of preoperative diagnosis and formulation of treatment plans, as mentioned within these papers.^[24,25]^ Additionally, this patient’s treatment process highlights the need for further research in order to establish clear diagnostic criteria for complications related to prostheses.

## 4. Conclusion

Complications associated with breast prosthesis reconstruction are intricate. The establishment of a definitive diagnosis for these complications is crucial in order to facilitate subsequent treatment. The examination and treatment processes employed in this case offer valuable insights toward achieving a more precise diagnosis of prosthesis-related complications and managing patients with complex medical histories.

## Acknowledgments

My sincere and hearty thanks and appreciations go to my supervisor, Dr Xiao Long, whose suggestions and encouragement have given me much insight into these translation studies. It has been a great privilege and joy to study under his guidance and supervision.

## Author contributions

**Conceptualization:** Yutong Yuan.

**Investigation:** Yutong Yuan.

**Resources:** Yutong Yuan.

**Writing – original draft:** Yutong Yuan, Fengzhou Du.

**Supervision:** Fengzhou Du.

**Data curation:** Yiding Xiao.

**Funding acquisition:** Fengzhou Du.

**Writing – review & editing:** Jiuzuo Huang, Xiao Long.

## References

[1] Mesdag V Régis C Tresch E. Nipple sparing mastectomy for breast cancer is associated with high patient satisfaction and safe oncological outcomes. J Gynecol Obstet Hum Reprod. 2017;46:637–42. 28690051 10.1016/j.jogoh.2017.07.003

[2] Colwell AS Taylor EM. Recent advances in implant-based breast reconstruction. Plast Reconstr Surg. 2020;145:421e–32e. 10.1097/PRS.0000000000006510 31985660

[3] Friedrich M Krämer S Friedrich D. Difficulties of breast reconstruction – problems that no one likes to face. Anticancer Res. 2021;41:5365–75. 34732406 10.21873/anticanres.15349

[4] Santanelli di Pompeo F Firmani G Stanzani E. Breast implants and the risk of squamous cell carcinoma of the breast: a systematic literature review and epidemiologic study. Aesthet Surg J. 2024;44:757–68. 38307034 10.1093/asj/sjae023

[5] Martin de Bustamante JM Mendoza A López-Muñoz S. A new face of fibrin-associated large B-Cell Lymphoma: Epstein–Barr virus-positive breast implant-associated diffuse large B-Cell Lymphoma. J Clin Med. 2023;12:3614. 37297811 10.3390/jcm12113614 PMC10253260

[6] Mikhaylov Y Weinstein B Schrank TP. Ketorolac and hematoma incidence in postmastectomy implant-based breast reconstruction. Ann Plast Surg. 2018;80:472–4. 29538000 10.1097/SAP.0000000000001409

[7] Hammond JB Kosiorek HE Cronin PA. Capsular contracture in the modern era: a multidisciplinary look at the incidence and risk factors after mastectomy and implant-based breast reconstruction. Am J Surg. 2021;221:1005–10. 32988607 10.1016/j.amjsurg.2020.09.020

[8] Vinsensia M Schaub R Meixner E. Incidence and risk assessment of capsular contracture in breast cancer patients following post-mastectomy radiotherapy and implant-based reconstruction. Cancers (Basel). 2024;16:265. 38254756 10.3390/cancers16020265 PMC10813520

[9] Stevens WG Calobrace MB Alizadeh K. Ten-year core study data for Sientra’s food and drug administration-approved round and shaped breast implants with cohesive silicone gel. Plast Reconstr Surg. 2018;141:7S–19S. 29595714 10.1097/PRS.0000000000004350

[10] Duteille F Perrot P Bacheley MH. Ten-year safety data for Eurosilicone’s round and anatomical silicone gel breast implants. Aesthet Surg J Open Forum. 2019;1:ojz012. 33791608 10.1093/asjof/ojz012 PMC7671289

[11] Kolasiński J Sorotos M Firmani G. BIA-ALCL epidemiology in an aesthetic breast surgery cohort of 1501 patients. Aesthet Surg J. 2023;43:1258–68. 37289985 10.1093/asj/sjad181

[12] Cordeiro PG Ghione P Ni A. Risk of Breast Implant Associated Anaplastic Large Cell Lymphoma (BIA-ALCL) in a cohort of 3546 women prospectively followed long term after reconstruction with textured breast implants. J Plast Reconstr Aesthet Surg. 2020;73:841–6. 32008941 10.1016/j.bjps.2019.11.064 PMC7247945

[13] Santanelli di Pompeo F Sorotos M Clemens MW. Mortality rate in breast implant surgery: is an additional procedure worthwhile to mitigate BIA-ALCL Risk? Aesthetic Plast Surg. 2023;47:914–26. 36376583 10.1007/s00266-022-03138-5 PMC10229447

[14] Jimenez RB Packowski K Horick N. The timing of acute and late complications following mastectomy and implant-based reconstruction. Ann Surg. 2023;278:e203–8. 35837894 10.1097/SLA.0000000000005574

[15] Krämer S Kümmel S Camara O. Partial mastectomy reconstruction with local and distant tissue flaps: review. Breast Care. 2007;2:299–306.

[16] Vorstenbosch J Chu JJ Ariyan CE. Clinical implications and management of non-BIA-ALCL breast implant capsular pathology. Plast Reconstr Surg. 2023;151:20e–30e. 10.1097/PRS.0000000000009780 PMC9797444 36194076

[17] de Kerckhove M Iwahira Y. Risk factors for capsular contracture: a retrospective study in immediate reconstruction versus delayed reconstruction. Plast Reconstr Surg Glob Open. 2020;8:e2864. 33133911 10.1097/GOX.0000000000002864 PMC7572041

[18] Kim HB Han HH Eom JS. Magnetic resonance imaging surveillance study of silicone implant-based breast reconstruction: a retrospective observational study. Plast Reconstr Surg Glob Open. 2023;11:e5031. 37305200 10.1097/GOX.0000000000005031 PMC10256406

[19] Lindenblatt N El-Rabadi K Helbich TH. Correlation between MRI results and intraoperative findings in patients with silicone breast implants. Int J Womens Health. 2014;6:703–9. 25114595 10.2147/IJWH.S58493 PMC4124066

[20] Gorczyca DP Gorczyca SM Gorczyca KL. The diagnosis of silicone breast implant rupture. Plast Reconstr Surg. 2007;120(7 Suppl 1):49S–61S. 10.1097/01.prs.0000286569.45745.6a 18090814

[21] Clemens MW Jacobsen ED Horwitz SM. 2019 NCCN consensus guidelines on the diagnosis and treatment of Breast Implant-Associated Anaplastic Large Cell Lymphoma (BIA-ALCL). Aesthet Surg J. 2019;39(Suppl_1):S3–S13. 30715173 10.1093/asj/sjy331

[22] Alotaibi S Hamadani M Al-Mansour M. Breast implant-associated anaplastic large cell lymphoma. clin Lymphoma Myeloma Leuk. 2021;21:e272–6. 33384263 10.1016/j.clml.2020.12.005

[23] Siminiak N Czepczyński R. PET-CT for the staging of breast implant- -associated anaplastic large cell lymphoma. Nucl Med Rev Cent East Eur. 2019;22:90–1. 31482564 10.5603/NMR.a2019.0015

[24] Santanelli Di Pompeo F Panagiotakos D. BIA-ALCL epidemiological findings from a retrospective study of 248 cases extracted from relevant case reports and series: a systematic review. Aesthet Surg J. 2023;43:545–55. 36441968 10.1093/asj/sjac312

[25] Paolini G Firmani G Briganti F. Assessment of risk factors for rupture in breast reconstruction patients with macrotextured breast implants. Aesthetic Plast Surg. 2023;47:517–30. 36229658 10.1007/s00266-022-03118-9 PMC10070228

